# All-Electrical Control of Compact SOT-MRAM: Toward Highly Efficient and Reliable Non-Volatile In-Memory Computing

**DOI:** 10.3390/mi13020319

**Published:** 2022-02-18

**Authors:** Huai Lin, Xi Luo, Long Liu, Di Wang, Xuefeng Zhao, Ziwei Wang, Xiaoyong Xue, Feng Zhang, Guozhong Xing

**Affiliations:** 1 Key Laboratory of Microelectronic Devices & Integrated Technology, Institute of Microelectronics, Chinese Academy of Sciences, Beijing 100029, China; linhuai@ime.ac.cn (H.L.); liulong@ime.ac.cn (L.L.); wangdi2021@ime.ac.cn (D.W.); zxf576@mail.ustc.edu.cn (X.Z.); wangziwei21@mails.ucas.ac.cn (Z.W.); zhangfeng_ime@ime.ac.cn (F.Z.); 2 University of the Chinese Academy of Sciences, Beijing 100049, China; 3 Department of Functional Material Research, Central Iron and Steel Research Institute, Beijing 100081, China; z3249985unsw@hotmail.com; 4 School of Microelectronics, University of Science and Technology of China, Hefei 230026, China; 5 State Key Laboratory of ASIC and System, School of Microelectronics, Fudan University, Shanghai 201203, China; xuexiaoyong@fudan.edu.cn

**Keywords:** spin-orbit torque, field-free magnetization switching, Fe_3_GeTe_2_, magnetoresistive random-access memory, non-volatile in-memory computing

## Abstract

Two-dimensional van der Waals (2D vdW) ferromagnets possess outstanding scalability, controllable ferromagnetism, and out-of-plane anisotropy, enabling the compact spintronics-based non-volatile in-memory computing (nv-IMC) that promises to tackle the memory wall bottleneck issue. Here, by employing the intriguing room-temperature ferromagnetic characteristics of emerging 2D Fe_3_GeTe_2_ with the dissimilar electronic structure of the two spin-conducting channels, we report on a new type of non-volatile spin-orbit torque (SOT) magnetic tunnel junction (MTJ) device based on Fe_3_GeTe_2_/MgO/Fe_3_GeTe_2_ heterostructure, which demonstrates the uni-polar and high-speed field-free magnetization switching by adjusting the ratio of field-like torque to damping-like torque coefficient in the free layer. Compared to the conventional 2T1M structure, the developed 3-transistor-2-MTJ (3T2M) cell is implemented with the complementary data storage feature and the enhanced sensing margin of 201.4% (from 271.7 mV to 547.2 mV) and 276% (from 188.2 mV to 520 mV) for reading “1” and “0”, respectively. Moreover, superior to the traditional CoFeB-based MTJ memory cell counterpart, the 3T2M crossbar array architecture can be executed for AND/NAND, OR/NOR Boolean logic operation with a fast latency of 24 ps and ultra-low power consumption of 2.47 fJ/bit. Such device to architecture design with elaborated micro-magnetic and circuit-level simulation results shows great potential for realizing high-performance 2D material-based compact SOT magnetic random-access memory, facilitating new applications of highly reliable and energy-efficient nv-IMC.

## 1. Introduction

Spintronic devices are attracting tremendous attention in the applications of the storage and processing of data by manipulating the spin rather than the charge of electrons, demonstrating the superiority of device and circuit level performance in data processing speed, scalability, and non-volatility, etc. [1,2]. The representative spin transfer torque magnetic random access memory (STT-MRAM) technology is being commercialized and applied to the high density data storage and neuromorphic computing as one of most typical non-volatile emerging memory technologies [2,3,4,5]. In 2012, the conceptual design of spin-orbit torque magnetic random access memory (SOT-MRAM) was proposed by L. Q. Liu et al. [6] with the faster magnetization switching speed (sub-ns) and the higher endurance (>10^10^) with mitigation of barrier layer degradation by separating the writing current flow through the spin-orbit coupling layer instead of the barrier layer of magnetic tunneling junction (MTJ) [6], i.e., the novel design splits the read and write path individually and avoids the interference and damage caused by the read and write current injected into the MTJ in STT-MRAM. Experimentally, the SOT-MRAM not only enhances the reliability and endurance but also reduces the energy consumption of the device operation, facilitating the highly reliable and time- and energy-efficient applications in the field of non-volatile memory and *nv*-IMC, as described in our previous work [1,7,8,9].

However, SOT-MRAM still bears the critical scientific and technical problems that need to be solved. Firstly, SOT-MRAM needs an external magnetic field to assist a deterministic switching in the writing process, which is difficult to enable due to the integration of the device into the standard complementary metal-oxide-semiconductor (CMOS) process [6,10]. Secondly, the traditional 2-transistor-1-MTJ (2T1M) cell structure utilizes the bipolar current to write different states. Due to the source degeneration of the metal-oxide-semiconductor field effect transistor (MOSFET), bipolar writing current is asymmetric, leading to the long write latency and low reliability of data storage [11,12]. In addition, during the reading process, the difference between the two resistance states of the MTJ can be characterized by tunnel magnetoresistance (TMR) ratio [13]. However, due to the limitations of materials and manufacturing processes, the TMR value that can be obtained at room temperature is relatively small [13], leading to the low sensing margin (SM) [14]. It is noted that a reading error occurs in the circuit if the sensing margin cannot overcome the process variation of the reading circuit, which, in turn, severely hurts the reading reliability performance.

In recent years, the rise of exploration and engineering in advanced 2D vdW materials, such as MoS_2_, WSe_2_, and Fe_3_GeTe_2_ (FGT), and 2D vdW antiferromagnets, etc., has provided a new materials platform for further study of spintronics [15,16,17,18,19,20,21,22,23,24,25,26,27]. FGT has high-quality surfaces, large coercivity, and perpendicular magnetic anisotropy (PMA) [28]. In addition, more systematic studies have reported on the enhancement of the Curie temperature (*T*_c_) of FGT [29,30,31] and the room temperature ferromagnetism of FGT with the gate modulation [32], ion doping [33,34], proximity coupling [35], exchange coupling effect [17], and atomic ratio engineering [36], demonstrating the excellent potential applications from the materials science and engineering performance perspectives. Encouragingly, the theoretically reported [37] and experimentally validated giant TMR values in 2D vdW FGT materials [38,39] significantly poise it as the cornerstone of MTJ devices aiming at the alleviation of read disturb and the enhancement of sensing margin in SOT-MRAM memory cell-circuit.

In the present work, we report a novel FGT/MgO/FGT heterostructure-based MTJ consisting of a magnetic free layer of 2D single FGT with PMA. By adjusting the ratio of the field-like torque to the damping torque coefficient (λ_FL_/λ_DL_) [40,41], the free layer magnetization switching direction of the MTJ can be determined by uni-polar writing current without an assistive external magnetic field [12,42]. Moreover, the TMR ratio of FGT/MgO/FGT heterostructure reaches up to 252% [43], enabling the improvement of the reading reliability of SOT-MRAM in comparison with the traditional CoFeB-based MTJ’s counterpart. Importantly, compared to the traditional SOT-MRAM memory cell structure of 2T1M, we developed a 3T2M memory cell with the self-reference feature to enhance the SM of read “0” from 26% to 132.6% (the sense voltage is boosted from 188.2 mV to 520 mV) and that of read “1” from 29.8% to 67.2% (the sense voltage is improved from 271.7 mV to 547.2 mV) [44], significantly alleviating the influence of process variation on the accuracy of the reading. Furthermore, employing such developed memory cell and an asymmetric pre-charge sense amplifier (PCSA), we successfully implemented a *nv*-IMC circuit upon AND/NAND and OR/NOR logic operations in sub-ns with ultra-low energy consumption of 57.53 fJ and 57.86 fJ, respectively, which is superior to the corresponding operations based on 2T1M SOT-MRAM and 6T-SRAM in terms of memory cell area, operation speed, and power consumption.

## 2. Results and Discussion

### 2.1. Proposed Device Structure and Parameters Optimization

**Figure 1:**
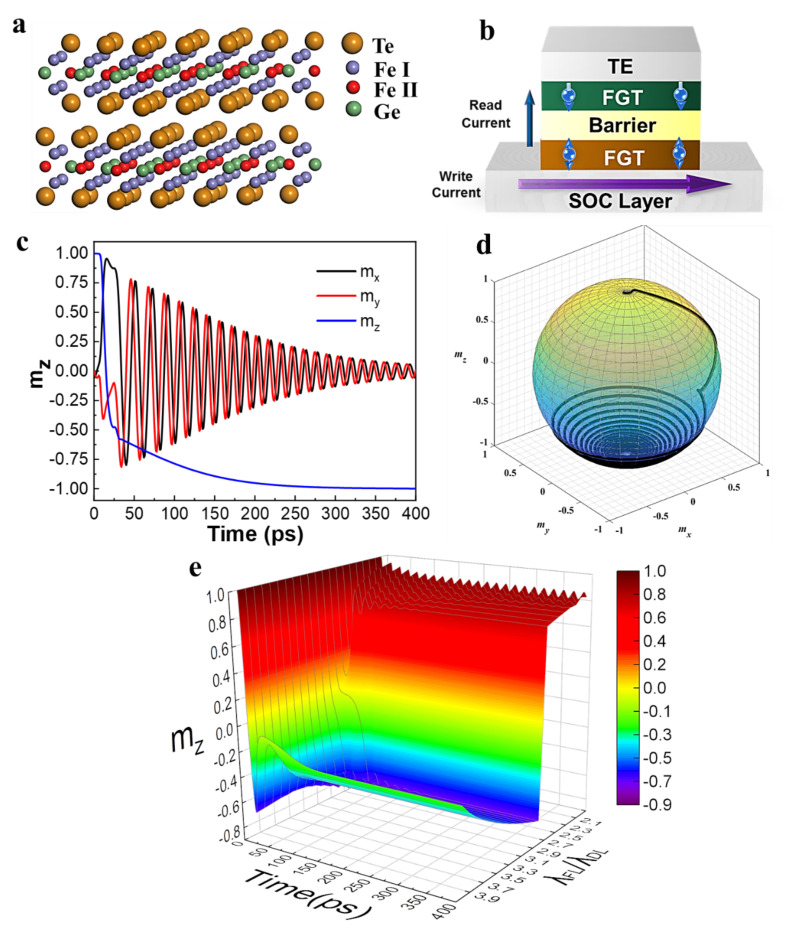
Proposed FGT-MTJ device: materials system, device structure, and magnetization switching dynamics. (**a**) The atomic crystal structure of FGT. (**b**) The basic FGT-MTJ structure. (**c**) Macro-spin simulation results of the time-dependent x-, y-, z-component magnetization (m_x_, m_y_, m_z_) in the perpendicular FGT-MTJ under all-electrical control (pulsed current of *J*_e_ = 7.9 × 10^7^ A/cm^2^ within 24 ps) without extra in-plane magnetic field. (**d**) Corresponding 3D m_z_ precession dynamic trajectories after the pulse current are applied with an optimal ratio of λ_FL_/λ_DL_ = 4. (**e**) The phase diagram of time dependent m_z_ switching with different λ_FL_/λ_DL_ ratios under *J*_e_ = 7.9 × 10^7^ A/cm^2^.

This study explores the single-layer 2D van der Waals material FGT with an atomic structure, as shown in Figure 1a. The lattice constants are a = b = 3.991 Å and c = 16.33 Å [45]. The adjacent layers of FGT are connected by weak van der Waals force; therefore, a 0.8 nm ultra-thin single layer structure can be obtained by mechanical exfoliation and transfer process or chemical vapor deposition method, with excellent interface flatness [22,46]. Importantly, upon the composition, doping, and/or bandgap engineering, such ultra-thin FGT still maintains the high magnetic anisotropy and high thermal stability, showing the unique advantages as a cornerstone of building blocks for nano-scale, highly reliable, and time- and energy-efficient spintronic devices [33,47]. Recently, the molecular beam epitaxy/chemical vapor deposition method is applied in the preparation of FGT [17,36], which further broadens the large-scale application prospects of FGT materials. Figure 1b shows the proposed FGT-based MTJ device structure in the present study. The MTJ pillars with dimension of 60 nm × 60 nm are utilized, including the top electrode (TE), ferromagnetic reference layer (RL), MgO spacer/barrier layer, ferromagnetic free layer (FL) and spin-orbit coupling (SOC) layer, in which the ferromagnetic RL and the ferromagnetic FL are composed of single layer FGT with appropriate composition, thickness, and interfacial exchange coupling engineering [17]. Based on the latest theoretical and experimental reports on the FGT materials system, the saturation magnetization (*M*_s_), effective anisotropy (*H*_k_), and Gilbert damping constant (*α*) etc. [32,48,49], basic magnetic parameters are adopted in the present work with the values, as listed in Table 1. The proposed MTJ is constructed with an easy magnetization direction perpendicular to the plane, i.e., PMA [13], which is conducive to the device minimization and the high-speed magnetization switching.

Most recently, the FGT system shows the intriguing properties in effective SOT applications because of considerable SOC strength, which can effectively convert the charge current into a spin current, resulting in a larger SOT [22,50]. Briefly, the writing current pulse is injected to the SOC layer during the writing process with the spin current generated due to the spin Hall effect in the SOC layer (e.g., Pt, Ta, and W, etc.) [51]. Then, the injected polarized current will produce a SOT on the ferromagnetic free layer, which is described by a field-like and a damping-like torque [52]. The deterministic magnetization switching without the assistance of an external magnetic field can be realized when the ratio of the field-like torque to the damping moment torque (λ_FL_/λ_DL_) reaches the optimal values [53]. A macro-spin simulation model was employed and performed to simulate the dynamic process of FL magnetization switching driven by current without external magnetic field with no involvement of Heisenberg exchange, demagnetization, and dipole interaction. Figure 1c,d illustrate the magnetization (m_x_, m_y_, m_z_) component time-dependent precession trajectory evolution of the FL magnetization under a *J*_e_ of 7.9 × 10^7^ A/cm^2^ (i.e., 94.9 μA current flowing through heavy metal/SOC track layer in dimension of 60 nm width and 2 nm thickness), which yields a considerably faster switching. During the application of an appropriate current pulse, the magnetization direction precesses with crossing the middle position from the initial top position. Concurrently, the current reaches the falling edge, and the magnetization direction of the FGT relaxes without the external current and gradually oscillates to the equilibrium position.

The realization of all-electrical control of SOT-MRAM involves the reciprocal optimization and iterative settings of key magnetic characteristics of materials and the electrical parameters of the device. In our work, a developed Verilog-A MRAM model and the commercial Cadence software with a foundry’s 55 nm process design kit (PDK) are utilized to radically explore the device performance [12]. The effect of adjusting λ_FL_/λ_DL_ on the z axis normalization magnetization m_z_ (m_z_ = *M*_z_/*M*_s_) of the ferromagnetic free layer in MTJ is shown in Figure 1e. As to FGT materials, when the ratio of λ_FL_/λ_DL_ is relatively small (<3), the m_z_ always oscillates on the one side of the initial magnetization direction and fails to switch the resistance state. On the contrast, when the ratio of λ_FL_/λ_DL_ is sufficiently large (≥3), the magnetization direction m_z_ passes through the *xy*-plane from the initial position to the other side, accomplishing a rapid switching without an external magnetic field, and relaxes at the final equilibrium position with intrinsic oscillations. Therefore, the deterministic writing is realized by modulating the λ_FL_/λ_DL_ ratio. Typically, there are some representative methodologies to modulate the ratio of λ_FL_/λ_DL_; for instance, by means of either the bulk Rashba channels enhancement or the interfacial spin accumulation, it is amenable to enhance the FL torque and boost the ratio of λ_FL_/λ_DL_ up to 4 [54]. By annealing at the proper temperature, the ratio of λ_FL_/λ_DL_ can be greatly enhanced due to the improvement of interfacial spin transparency [55]. The λ_FL_/λ_DL_ ratio can also be adjusted appropriately by partial oxidation or hydrogenation at the ferromagnetic and SOC layer interfaces [56,57]. Moreover, the Ostered field produced by the current also has a certain enhancement effect on FL torque, which can help to improve the ratio moderately [41]. It is worthy to note that although a larger λ_FL_/λ_DL_ promotes a faster magnetization switching and shortens the write delay, the peak value of m_z_ and the oscillation interval after the switching are closer to the middle position in the plane (m_z_ = 0); in the actual process, it is more susceptible to interference and mis-writing.

**Figure 2:**
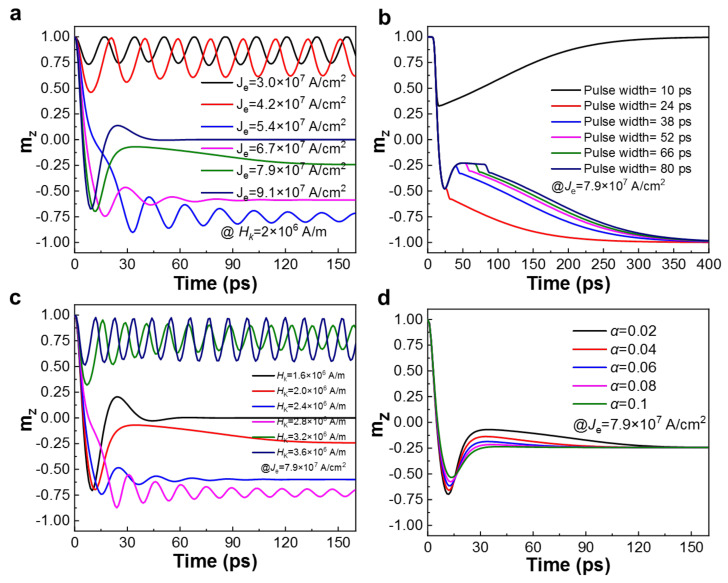
Dependence of magnetization switching of SOT-MTJ on different parameters. The effect of different *J*_e_ (**a**) and pulse width (**b**) on the magnetization switching. (**c**) The magnetization switching of proposed FGT-MTJ device with various *H*_K_ and the field-free switching of materials with different Gilbert damping constant (*α*) under *J*_e_ of 7.9 × 10^7^ A/cm^2^ (**d**).

Figure 2a,b show the impact of the current density *J*_e_ and pulse width of the write current on the field-free switching of the MTJ, respectively. Clearly, upon the exertion of a field-like torque on the ferromagnetic FL when the write current flows through the SOC layer, the magnetization reversal characteristic’s dependence on the write current density is consistent with the results obtained from Figure 1e. In contrast, when *J*_e_ is lower than the magnetization switching threshold current density, the m_z_ oscillates on the side of the initial position [58]. Nevertheless, the deterministic switching is achieved once the threshold current density is exceeded. Similarly, the excessive current density will also negatively affect the write reliability [59].

On the other hand, the pulse width of the current exhibits a critical influence on field-free switching. Specifically, the m_z_ relaxation starts with the falling edge of the pulse current. Consequently, the change of m_z_ position in Figure 2b with a pulse width of 10 ps is shorter than the time that is required for the magnetization switching through the middle position, causing m_z_ to relax on the initial side and leading the failure of desired complete magnetization switching. Although the pulse width, which is longer than 38 ps, realizes the reversal of m_z_, the resting position is close to the *xy*-plane, which results in a longer relaxation time eventually, and is simultaneously more susceptible to interference and errors. Upon application of pulse width 24 ps, the current duration is longer than the time required for the complete magnetization reversal. In this case, the m_z_ achieves the desired deterministic switching. Note that when the pulse width is longer than the time required for the threshold, the increase of pulse width cannot further improve the switching result but increases the relaxation time and energy consumption, which is not favorable to the practical applications. Therefore, based on the above implementation with complementary investigations, the pulse profile can be preferably optimized with a design enable process, i.e., the width matches the m_z_ switching time to achieve minimized delay, low energy consumption, and high reliability, etc.

The characteristics of the ferromagnetic material itself in MTJ also determine the performance of the proposed device. Table 2 summarizes the FGT-based MTJ with different *H*_K_ and the corresponding threshold current (*I*_c_), writing latency, and power consumption for the deterministic magnetization switching. It clearly demonstrates that the tailored materials with a larger *H*_K_ warrant devices to be functional with a shorter writing latency. At the same time, a larger threshold current is required to achieve a field-free deterministic magnetization switching, which causes an increase in power consumption. On the contrary, as to the FGT with a smaller *H*_K_ value, a lower writing current is required to complete the deterministic magnetization switching. However, the longer the switching time, the longer the write pulse required to be applied, resulting an unavoidable increased write energy consumption. Therefore, the FGT material with *H*_K_ = 2 × 10^6^ A/m is favorable to the application of low-latency and high-energy efficiency field-free SOT-MRAM [32,60], which is in line with the recent work that provides the experimental guidance for the modulation of the *K*_u_ value in FGT [61].

The characteristic magnetization switching dynamics of FGT-based MTJs with different *H*_K_ values driven by a direct current of 94.9 μA are shown in Figure 2c. For the case of *H*_K_ = 2.4 × 10^6^ A/m, the write current requires to be close to its threshold current of 92.7 μA, which executes a complete magnetization switching without external field application, and m_z_ is always on the opposite side of the initial position. For the larger *H*_K_ value of 3.2 × 10^6^ A/m, the same write current density is lower than its critical current, which is not enough to reverse m_z_. Therefore, m_z_ always oscillates on the initial side and cannot achieve a deterministic switching. For the case that the *H*_K_ value is small (*H*_K_ < 2 × 10^6^ A/m), the applied current is greater than its threshold current, leading to the faster switching of magnetization. However, it also results in the damping oscillation of m_z_ and finally approaches the in-plane position. Consequently, the field-free SOT MRAM requires a reasonable write current interval to be designed according to material characteristics.

The Gilbert damping constant (*α*) is a key physical index of magnetic FL, revealing the energy dissipation path of a given magnetic system. Physically, it is influenced by both the intrinsic material properties and many extrinsic sources. In particular, ultra-thin magnetic layers in MTJ (e.g., 2D vdW FGT) and spin pumping from adjacent layers would tailor *α* significantly [62,63,64]. Figure 2d reveals the switching phenomenon with different *α* values under *J*_e_ = 7.9 × 10^7^ A/cm^2^. For a smaller value of *α*, the MTJ has a faster magnetization switching speed. Therefore, the pulse width limited by the critical switching time as well as the write energy consumption is reduced at the same time.

### 2.2. 3T2M Cell Structure

**Figure 3:**
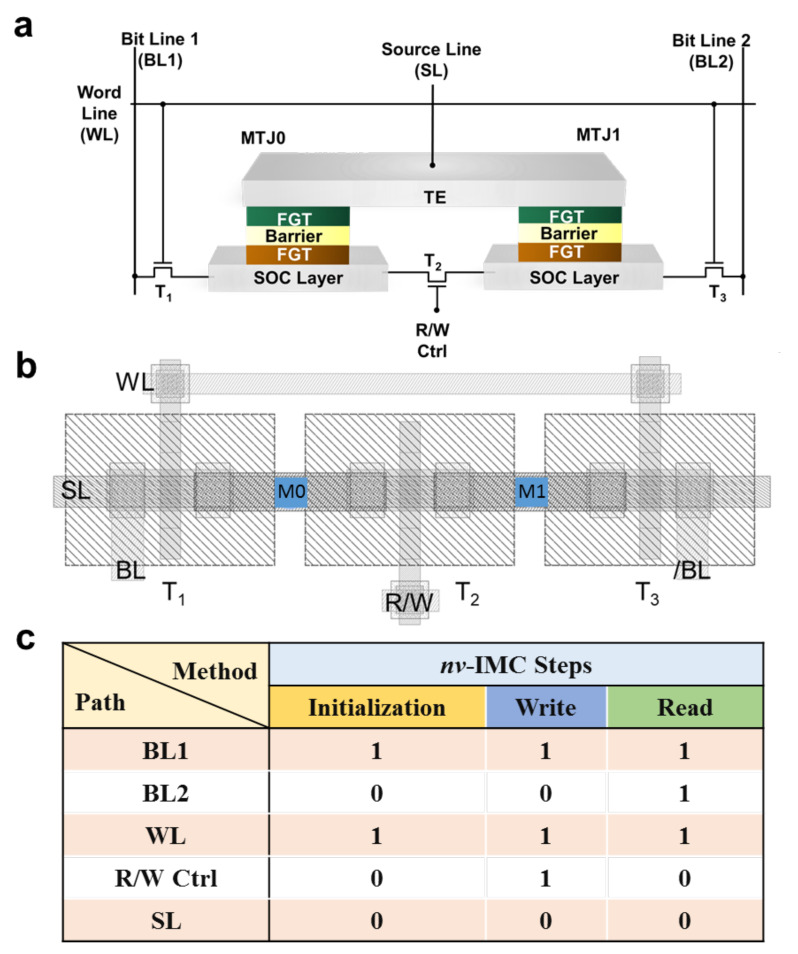
The storage cell structure and corresponding operation scheme. (**a**) 3T2M memory cell structure diagram. (**b**) The layout of 3T2M structure. (**c**) 3T2M memory cell initialization, writing, reading, and logical operations.

In order to increase the reliability of the device reading and IMC process [65], a 3-transistor-2-MTJ (3T2M) self-reference cell structure is proposed based on the FGT-MTJs, which not only plays the role in executing as a non-volatile memory but also realizes the reconfigurable IMC logic operation. As shown in Figure 3a, the structure is composed of three transistors (T1/T2/T3) and two FGT-MTJs (MTJ0/MTJ1). Figure 3b illustrates the layout of 3T2M cell structure, where the gates of the transistors T1/T3 are connected to the word line (WL), controlling the reading and writing process of the memory cell and the on/off state of transistors during the operation. T2 is connected to the reading and writing control line (R/W Ctrl) to separate the writing and the reading path individually. Top terminals of the MTJs are connected to the source line (SL) as the path for reading and storing calculation results. The main operations involved in the memory cell and the corresponding voltage bias state are shown in Figure 3c. An initialization step needs to be performed when the memory cells are used for the first time. At this step, the current passes through MTJ0 and MTJ1 from the opposite direction, and the two MTJs are initialized to the complementary states by the spin-transfer torque (STT) effect [66]. Here, the low- and high-resistance states of MTJ are set as “0” and “1”, respectively. Therefore, if the initialization current drives the magnetization of FL of MTJ0 parallel to the RL, the MTJ0 is “0”, and MTJ1 is “1”.

The difference between memory cell write operation and initialization step is that R/W Ctrl is at a high level in the former operation, where T2 is turned on, and current flows through the SOC layer, which has a smaller resistance. The spin current is generated through the spin-orbit coupling effect, which injects to the FGT free layer of MTJ. The m_z_ in the MTJ0/MTJ1 free layer switches to the opposite state of the initial state at the same time to achieve the purpose of writing information, when the λ_FL_/λ_DL_ and current density meet the deterministic magnetization switching condition, as addressed in previous sections.

**Figure 4:**
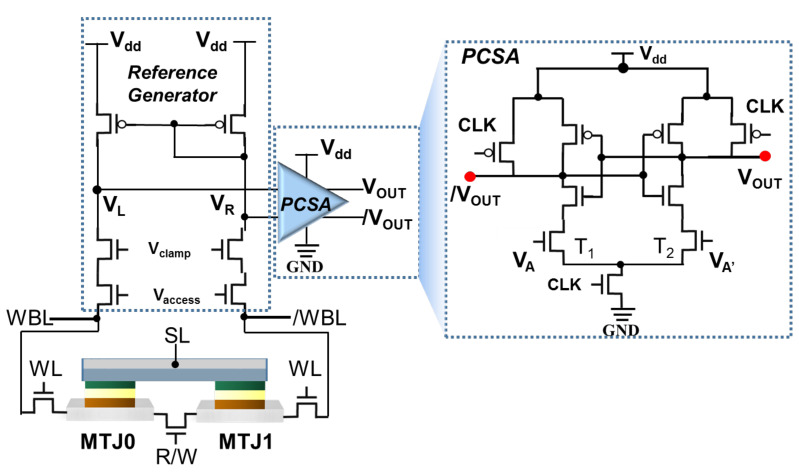
Read/write circuit architecture for 3T2M FGT-MTJ memory cell. Schematic diagram of the logic operation sub-circuit structure with an asymmetric PCSA configuration.

In order to significantly enhance the SM and further improve the reliability of the proposed architecture, our work utilizes the 3T2M self-reference cell structure reference generator and the symmetrical PCSA to construct the read and write circuit of the memory cell, as depicted in Figure 4. V_access_ and WL are employed to select the memory cell in the array, and a clamping voltage V_clamp_ is applied to the data cell and the self-reference cell. WBL = V_dd_ and $\bar{\mathrm{WBL}}$ = 0 are activated to generate write pulses for operation during initialization and writing process. MTJ0 is used as the data storage terminal of the memory cell. Then, MTJ1, which stores the complementary state, is used instead of R_ref_ as the reference resistance of MTJ0 during the reading process. Therefore, SM = $\frac{\left|V_{\mathrm{MTJ}0}{-V}_{\mathrm{MTJ}1}\right|}{V_{\mathrm{MTJ}1}}\times 100\%$ which is larger than the value presented from the traditional SM regime. Different values of V_sense_ and V_ref_ are generated in the two branches of the reference generator, V_L_ and V_R_, respectively, owing to the ΔR between MTJ0 and MTJ1, when the reading circuit is activated. Consequently, the PCSA is pre-charged to V_dd_ in the initial stage. After the read enable signal CLK comes, the V_L_ and V_R_ voltages are set as the gate voltage of transistor of PCSA (T1 and T2 in the Figure 4) to control the discharge speed of the PCSA branch, which will generate complementary output results V_out_ and $\bar{V_{\mathrm{out}}}$, where V_out_ outputs a high (low) voltage level, facilitating the MTJ0 storage state “1” (“0”) to realize the read operation in the memory cell.

**Figure 5:**
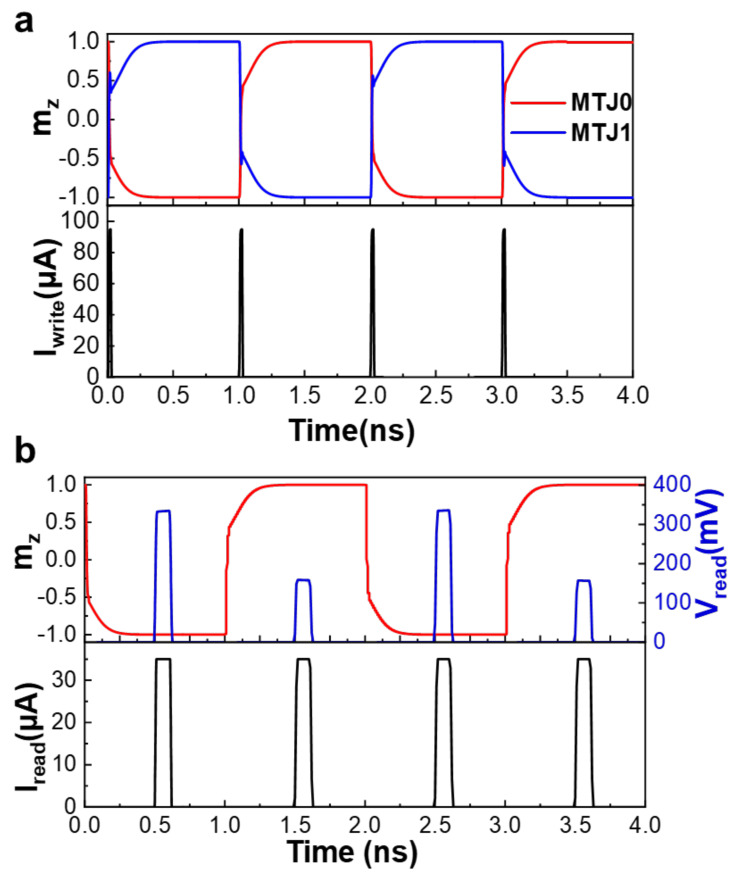
Writing and reading process for 3T2M storage cell. (**a**) The switching of magnetization of MTJ0 and MTJ1 with successive I_write_ applied to the writing path. (**b**) The read pulse I_read_ is applied to the MTJ after the writing operation, and the read voltage and V_read_ is generated in MTJ0 with difference m_z_ state.

In order to validate the performance of the proposed device cell, the Cadence with A foundry 55 nm CMOS PDK is utilized to simulate the FGT-based MTJs switching and the logic operation properties of the 3T2M memory cell. [7] As shown in Figure 5a, the writing process of MTJ0 and MTJ1 is systematically conducted. Briefly, MTJ0 is switched from the original “1” state to the “0” state, and MTJ1 is switched from the original “0” state to the “1” state when the circuit applies the first write pulse I_write_ to the write path of 3T2M cell, and, consequently, both MTJs realize switching without any external magnetic field at the same time in a sub-nanosecond. If we need to change the stored binary state after the first switching, the current pulses are to be injected in the same direction again, and then the free layer in the MTJ will repeat the above switching process oppositely. Noted that each write pulse can switch the magnetization state of the free layer in the MTJ to the opposite side, thereby making the controllable uni-polar field-free switching of SOT-MRAM.

Upon application of a read pulse current (I_read_) to the memory cell, enabling the generation of the read voltage (V_read_), which corresponds to the resistance state in the MTJ after the writing operation for the 3T2M memory cell, the simulation results of Figure 5b demonstrate that the V_read_ is generated by MTJ0 for different storage states, when I_read_ = 35 μA. Note that the peak value of I_read_ is smaller than that of the write current and the initialization current. Therefore, the smaller read current hardly affects the m_z_ position as shown in Figure 5b. The m_z_ position precesses to close to −1 and V_read_ = 333 mV in the first reading pulse, which means that the magnetization direction of the ferromagnetic free layer in the MTJ is parallel to the magnetization direction of the ferromagnetic reference layer, reflecting a low resistance of MTJ0. At this time, the data stored in the MTJ0 are “0”. Then, the m_z_ position precesses to close to 1, and V_read_ = 158 mV, indicating that the magnetization direction of the ferromagnetic free layer in the MTJ is anti-parallel to that one of the ferromagnetic reference layer, and the resistance of the MTJ is relatively high. In turn, the data stored in the MTJ0 are a binary number “1”.

### 2.3. Read and Write Circuits with Elaborated Performance

In the traditional 1T1M STT-MRAM or 2T1M SOT-MRAM, the researchers generally employ the reference resistor *R*_ref_ to distinguish the state of the data stored in the memory cell [67,68].

(1) $$R_{\mathrm{ref}}=\frac{1}{2}\left(R_{p}+R_{\mathrm{ap}}\right)$$

where *R*_p_ and *R*_ap_ are the resistance of the MTJ when the magnetization of the reference layer and the free layer are parallel and anti-parallel, respectively. When the read voltage V_read_ is greater (lesser) than the reference voltage V_ref_, the low (high) level state is read through the sensitive amplifier, standing for “1” (“0”). The reliability of proposed method is based on the TMR of MTJ, where TMR = (*R*_ap_ − *R*_p_)/*R*_p_; the larger the TMR value, the larger the read SM, where SM =$\frac{\left|V_{\mathrm{read}}{-V}_{\mathrm{ref}}\right|}{V_{\mathrm{ref}}}\times 100\%$, thus the higher the read reliability that can be accomplished. However, the TMR based on traditional ferromagnetic materials such as CoFeB with PMA is only between 50% and 120% [13], with a smaller SM between V_read_ and V_ref_. As a result, the process fluctuations are more likely to cause the misreading of the MTJ states and further deteriorate the reliability. Based on the latest experimental and theoretical calculation reports [39,43], the moderate TMR ratio value of 250% is implemented in the present simulations work. 

**Figure 6:**
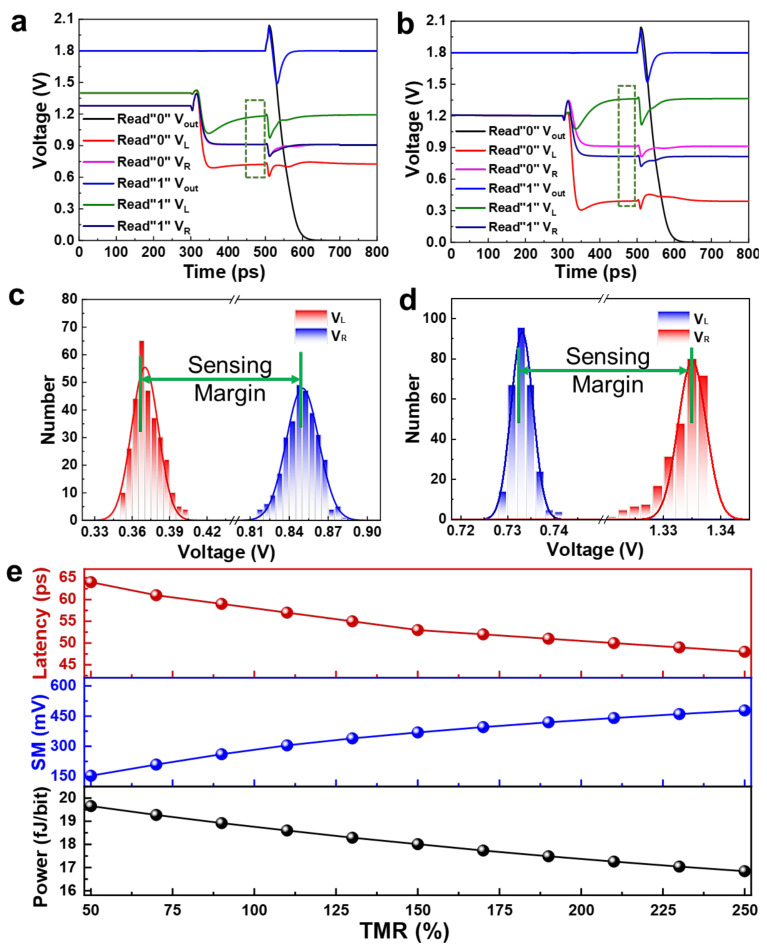
The reading performance of 2T1M and 3T2M SOT-MRAM. (**a**) The 2T1M and (**b**) 3T2M SOT-MRAM read result, “1” (blue) and “0” (black), respectively. The dynamic process of their branch voltage V_L_ and V_R_. The red dash frames show the SM, respectively. (**c**,**d**) The Monte Carlo simulation result of V_L_ and V_R_ in reading “0” (**c**) and “1” (**d**) of 3T2M SOT-MRAM. € The TMR of MTJ effects on latency (upper panel), SM (middle panel), and power consumption (lower panel) in the reading process of a 3T2M cell. (**e**) Impact of TMR on the reading performance of 3T2M structure.

The contrastive features of the reading process between 2T1M and 3T2M cell structures are depicted in Figure 6a,b. Firstly, V_access_ and WL are turned on in the circuit at 300 ps, and V_L_ and V_R_ output different read voltages. Then, CLK pulse is applied at PCSA at 500 ps, and PCSA differentially amplifies V_L_ and V_R_ to output the data stored in the MTJ0. According to the simulation results, the SM of 2T1M is 26% and 29.8% for read “1” and “0”, respectively, and correspondingly, the SM of 3T2M reaches 132.6% and 67.2%, which is much higher than the former, when the circuit reaches a steady state. It also shows that 3T2M has higher reading accuracy, facing process fluctuation or external interference. In order to further confirm the tolerance of 3T2M and reference generator circuits to process fluctuations, we added a change of *σ* = 5% to the transistor and carried out a 300-point Monte Carlo simulation for V_L_ and V_R_. The distribution results of Figure 6c,d show that, no matter if it is reading “1” or reading “0”, a higher SM ensures that the voltage values of V_L_ and V_R_ without overlap in the process fluctuations, which effectively avoiding errors during reading.

Delay, reading margin, and power consumption of the device are all important parametric indexes, and the TMR of the MTJ bear an important impact on the above performances. The upper panel of Figure 6e shows the influence of MTJ TMR on the reading performance of 3T2M structure. According to the latest reports, the TMR of traditional CoFeB/MgO/CoFeB perpendicular magnetic tunnel junction, i.e., *p*-MTJ devices is between 50% and 150%, while the TMR of 2D vdW materials based on MTJs, such as FGT/MgO/FGT, can reach 160% or even 252% [38,42]. According to the simulation results, it is found that, as the device TMR increases, the read delay of the device tends to be lower. Compared to 50% TMR with a latency of 24.57 ps, the delay is as low as 21.05 ps when TMR reaches to 250%, which is more beneficial to the fast reading and computing operations. The increase in the sensing margin provides a higher process tolerance for devices with higher TMR, as revealed from the results in the middle plane of Figure 6e, which improves the reliability of the reading. At the same time, the power consumption of the 3T2M structure with TMR = 250% is 16.84 fJ/bit, which is 15% less than the power consumption of 19.66 fJ/bit when TMR = 50%, as depicted in the lower panel of Figure 6e, illustrating the potential of low-power operation. Therefore, the 3T2M devices based on 2D vdW with enhanced TMR project a high potential in high-reliability, low-latency, and high-energy efficiency *n*v-IMC applications from practical implementation perspectives.

### 2.4. Non-Volatile In-Memory Computing

The computing system based on the traditional “von Neumann” architecture separates storage and computing, resulting in a “memory wall” effect, causing a series of problems such as limited bandwidth, reduced computing speed, and increased power consumption [69]. Therefore, in order to solve the above the problems, researchers use non-volatile memory such as resistive random access memory (RRAM) and MRAM to build IMC systems [7,70].

**Figure 7:**
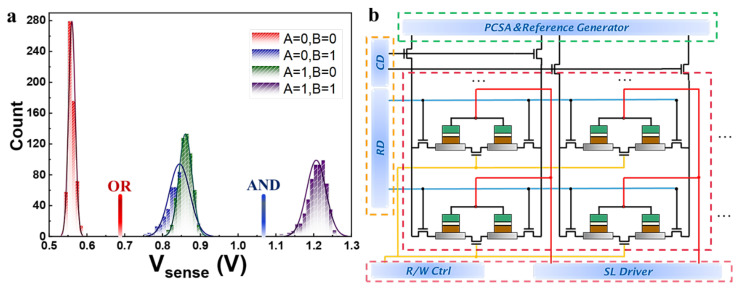
The implication of in-memory logic computation (**a**). Monte Carlo simulation distribution result of the sensing voltage for storing different data in the memory cell (**b**).

In the present work, the developed 3T2M FGT-MTs cells combined with an asymmetric pre-charge sense amplifier (PCSA) are designed to achieve a high-speed, low-power, and non-volatile memory IMC circuit. The logic operation circuit is shown in Figure 7b, which consists of a memory array, a voltage reading circuit, and an asymmetric sense amplifier. Any two of 3T2M self-referenced memory cells (A/A′ and B/B′) in the same column can be selected by their respective word lines for calculation in the SOT-MRAM sub-array. Two memory cells have four storage states, namely “AB” = 00,01,10,11, corresponding to three voltage states V_L_, namely “V_L_” = V_00_, V_01,10_, V_11_. Using Monte Carlo simulation, the distribution results of the sensing voltage (V_sense_) for the corresponding three states can be obtained, as shown in Figure 7a. Due to the sufficient SM, there is no overlap between the three types of output voltage distributions. Therefore, the circuit structure can avoid reading errors during computations.

AND/NAND Boolean logic: the transistors (T1 and T2 shown in Figure 4) of the asymmetric sensitive amplifier are set to different sizes during AND/NAND operation. T1: L = 60 nm, W = 120 nm, T2: L = 60 nm, W = 180 nm. When V_L_ = V_00_, V_L_ is less than V_R_, because of the complementary resistance state. Therefore, the discharge speed of the driving transistor T1 is less than T2, and V_out_ outputs low voltage level “0”. On the contrast, the discharge speed of the driving transistor T1 is greater than T2, when V_sense_ = V_11_, V_L_ > V_R_, and V_out_ = “1”. If V_sense_ = V_01,10_, two branch voltage V_L_ is equal to V_R_. The discharge speed of transistor T1 is lower than T2, because of channel width W_T1_ < W_T2_, and finally V_out_ outputs “0”. Realize A AND B operation, and $\bar{V_{\mathrm{out}}}$ output A NAND B operation result.

OR/NOR Boolean logic: The computation circuit adopts different design methods to change the transistor size of the asymmetric PCSA, among which T1: L = 60 nm, W = 180 nm, T2: L = 60 nm, W = 120 nm. This method ensures that the discharge speed of transistor T1 is faster than T2, when V_sense_ = V_01,10_ and V_L_ = V_R_, and because W_T1_ > W_T2_ and V_out_ outputs “1”. Therefore, realizing OR/NOR IMC result output by V_out_ and $\bar{V_{\mathrm{out}}}$. Furthermore, based on the output of AND/NAND and OR/NOR, different combinations of logic gates can be used to further implement logic operations such as XOR and XNOR, etc [71].

In the Cadence simulation software, we simulate the delay and average energy consumption for different MRAM and SRAM writes and logic operations, as shown in Table 3. Compared with the traditional CoFeB structure of MTJ, the 3T2M SOT-MRAM structure based on FGT material has higher speed, lower power consumption, and relative low cell size, which is mainly due to the advantages of FGT in magnetocrystalline anisotropy and TMR. The table further quantifies the advantages of the 3T2M structure over the traditional 2T1M structure in the same material system. It not only achieves faster logic operations but also reduces the writing speed and power consumption to 50% than 2T1R. Correspondingly, we compared with 6T-SRAM under the same technology node, and the result showed that 3T2M structure has lower energy consumption during the logic AND/OR operation in the memory, and the area of the memory cell is much smaller than the area occupied by 6T SRAM. It can also be compared in terms of speed.

## 3. Conclusions

In summary, we make full use of the advantages of 2D FGT materials and propose a 3T2M storage cell based on the FGT-based MTJ structures. The field-free switching can be achieved by modulating proper λ_FL_/λ_DL_ with modulation of various parameters, such as Ku and α, etc. The sensing margin of 3T2M is increased by 201.4% and 276% for reading “1” and “0” over that of the traditional 2T1M cell. Furthermore, the basic cell structure combined with asymmetric PCSA can realize basic AND/NAND and OR/NAND IMC Boolean operation with high speed and low power consumption. With extended and generalized 2D vdW materials [72] including 2D vdW antiferromagnets with the joint effect of Dzyaloshinskii–Moriya interaction and spin current for deterministic and fast switching [73], the proposed 3T2M MTJs cell structure is promising for more applications in the field of compact spintronic devices and *nv*-IMC.

## Figures and Tables

**Table 1:** The simulation parameters for 3T2M FGT-MTJ.

Parameter	Description	Value
*M_s_*	Saturation magnetization	1.7 × 10^5^ A/m
*H_K_*	Effective anisotropy	2.0 × 10^6^ A/m
*α*	Gilbert damping constant	0.02
*λ_FL_/λ_DL_*	Ratio of FL torque to DL torque	4
*θ_SH_*	Spin Hall angle of heavy metal	−0.3
*ρ_HM_*	Resistivity of heavy metal	200 μΩ·cm
*t_BL_*	Thickness of barrier layer	0.85 nm
PhiBas	Barrier potential height	0.4 eV
RA	Resistance–area product	10 μΩ·cm^2^
*A*_MTJ_	MTJ area	60 nm × 60 nm
TMR_0_	TMR ratio at zero bias	250
*W* × *L* × *t*_HM_	Heavy metal dimension	120 nm × 60 nm × 2 nm

**Table 2:** Tabulated magnetization switching threshold current, writing latency and power consumption of FGT-based MTJ with different magnetic anisotropy (*H*_k_).

*H*_K_ (A/m)	I_c_ (μA)	Latency (ps)	Power (fJ/bit)
1.6 × 10^6^	59.4	75	4.47
2.0 × 10^6^	75.6	61	4.51
2.4 × 10^6^	92.7	54	4.80
2.8 × 10^6^	113.6	53	5.65
3.2 × 10^6^	141.5	72	9.28
3.6 × 10^6^	No switching		

**Table 3:** Benchmark and comparison of the performance among different storage cells.

Device	Operation	Cell Area	Avg. Latency (ps)	Avg. Power Consumption (fJ/bit)
6T SRAM	Write	140 F^2^	57	5.61
	AND/NAND		22	71.37
	OR/NOR		19.25	71.48
2T1M SOT-MRAM (CFB)	Write	69 F^2^	218	56.62
	AND/NAND		50.75	118.19
	OR/NOR		43.5	117.68
3T2M SOT-MRAM (CFB)	Write	82.5 F^2^	200	35.53
	AND/NAND		39.25	93.55
	OR/NOR		48.25	93.80
3T2M SOT-MRAM (FGT)	Write	82.5 F^2^	24	2.47
	AND/NAND		35	57.53
	OR/NOR		45.25	57.86
